# Risk stratification of thyroid nodules with Bethesda category III results on fine-needle aspiration cytology: The additional value of acoustic radiation force impulse elastography

**DOI:** 10.18632/oncotarget.13685

**Published:** 2016-11-29

**Authors:** Chong-Ke Zhao, Hui-Xiong Xu, Jun-Mei Xu, Cheng-Yu Sun, Wei Chen, Bo-Ji Liu, Xiao-Wan Bo, Dan Wang, Shen Qu

**Affiliations:** ^1^ Department of Medical Ultrasound, Shanghai Tenth People's Hospital, Ultrasound Research and Education Institute, Tongji University School of Medicine, Shanghai 200072, China; ^2^ Thyroid Institute, Tongji University School of Medicine, Shanghai 200072, China; ^3^ Shanghai Center for Thyroid Diseases, Shanghai 200072, China; ^4^ Department of Endocrinology & Metabolism, Shanghai Tenth People's Hospital, Tongji University School of Medicine, Shanghai 200072, China

**Keywords:** thyroid nodules, fine-needle aspiration cytology, Bethesda category III, ultrasound, acoustic radiation force impulse elastography

## Abstract

To assess the value of conventional ultrasound, conventional strain elastography (CSE) and acoustic radiation force impulse (ARFI) elastography in differentiating likelihood of malignancy for Bethesda category III thyroid nodules. 103 thyroid nodules with Bethesda category III results on fine-needle aspiration cytology (FNAC) in 103 patients were included and all were pathologically confirmed after surgery. Conventional ultrasound, CSE and ARFI elastography including ARFI imaging and point shear wave speed (SWS) measurement were performed. Univariate and multivariate analyses were performed to identify the independent factors associated with malignancy. Area under the receiver operating characteristic curve (Az) was calculated to assess the diagnostic performance. Pathologically, 65 nodules were benign and 38 were malignant. Significant differences were found between benign and malignant nodules in ARFI. The cut-off points were ARFI imaging grade ≥ 4, SWS > 2.94 m/s and SWS ratio > 1.09, respectively. ARFI imaging (Az: 0.861) had the highest diagnostic performance to differentiate malignant from benign nodules, following by conventional ultrasound (Az: 0.606 - 0.744), CSE (Az: 0.660) and point SWS measurement (Az: 0.725 - 0.735). Multivariate logistic regression analysis showed that ARFI imaging grade ≥ 4 was the most significant independent predictor. The combination of ARFI imaging with point SWS measurement significantly improved the specificity (100% vs. 80.0%) and positive predictive value (100 % vs. 72.9%) in comparison with ARFI imaging alone. ARFI elastography is a useful tool in differentiating malignant from benign thyroid nodules with Bethesda category III results on FNAC.

## INTRODUCTION

Patients with suspicious thyroid nodules on ultrasound (US) are usually advised to undergo fine-needle aspiration cytology (FNAC). US-guided FNAC is a cost-effective and widely-used method to differentiate thyroid nodules with a diagnostic accuracy of 62% to 85%, which reduces the risk of unnecessary surgery for benign nodules [1–5]. The Bethesda System for Reporting Thyroid Cytopathology (BSRTC) has standardized the FNAC results and facilitated effective communication among clinicians, radiologists and pathologists [6]. However, cytological examination cannot replace the final pathological diagnosis on account of sampling errors [7]. The Bethesda category III classification, that is, atypia of undetermined significance (AUS) / follicular lesion of undetermined significant (FLUS), has remained ambiguous concerning the risk of malignancy and guidelines for management [3, 8]. Bethesda category III nodules (i.e. AUS/FLUS nodules) usually account for less than 7% of FNAC results and the malignancy rate is 5%–15% [9]. However, there are varying reports citing the incidence rate and the risk of malignancy, ranging from 3% to 20% [10, 11] and 5% to 48% [9, 12–14].

The uncertain malignancy risk of AUS/FLUS nodules always leads to uncertainty in subsequent treatment planning. To solve this issue, a lot of studies have investigated risk factors associated with malignancy in AUS/FLUS nodules, including clinical factors (sex, age, history of radiation and family history) [13], US findings [4, 15], elastography findings [7, 16], cytology subclassifications [3, 17], molecular mutational analyses [3], repeated FNAC [8, 18], core-needle biopsy (CNB) [17] and intraoperative frozen sections (FS) [2]. The recommended management for nodules with Bethesda category III result is repeat FNAC 3 month after first FNAC [6]. Surveillance or diagnostic surgery may also be advised, depending on clinical risk factors, US pattern, and patient preference [9]. In a recent prospective study, 48.6% of initial Bethesda category III nodules persisted as category III on repeat FNAC [8, 11], arguing against the role of repeat FNAC. Meanwhile, patients are often reluctant to undergo repeat FNAC.

Recently, US elastography has gained increasing attention for diagnosis of thyroid nodules. Conventional strain elastography (CSE) is helpful in differentiating malignant from benign thyroid nodules by enabling measurement of tissue deformation in response to compression and displaying tissue stiffness [19], whereas it is limited by factors such as lack of quantitative information and low reproducibility [20]. Acoustic radiation force impulse (ARFI) elastography has been introduced in recent years, in which the tissue is mechanically excited under short-duration acoustic pulses from the transducer which propagate in a perpendicular direction. Qualitative assessment of stiffness is achieved by estimating tissue displacement (i.e. ARFI imaging) under the acoustic pulses and quantitative assessment is achieved by measuring transverse shear wave propagation speed (i.e. point shear wave speed [SWS] measurement) [21]. ARFI elastography has showed improved diagnostic accuracy in comparison with conventional US and CSE [22, 23], which is also more reproducible and operator-independent [24].

Until present, no study has been performed to evaluate the usefulness of ARFI elastography for diagnosis of AUS/FLUS thyroid nodules. It was hypothesized that ARFI elastography might be a useful tool for malignancy stratification of AUS/FLUS thyroid nodules. To confirm this hypothesis, the diagnostic performance of ARFI elastography in diagnosis of AUS/FLUS nodules was prospectively evaluated and the possible predictors for malignancy were analyzed.

## RESULTS

### Surgical pathology

**Figure 1:**
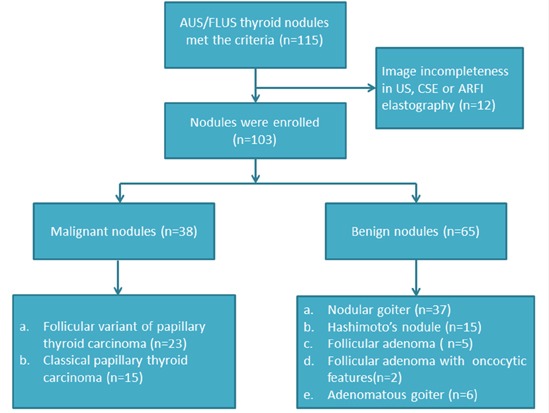
The flowchart of selection of the patients with thyroid nodules

There were 65 (63.1%) benign nodules and 38 (36.9%) malignant nodules on pathology after surgery. The benign nodules included nodular goiters (n=37), Hashimoto's nodules (n=15), follicular adenomas (n=5), follicular adenomas with oncocytic features (n=2), and adenomatous goiters (n=6). The 38 malignant nodules were all papillary thyroid cancers (PTCs), of which 21 (55.2%) nodules were microcarcinomas (Figure 1).

### Basic characteristics, US, CSE, and ARFI elastography

**Figure 2:**
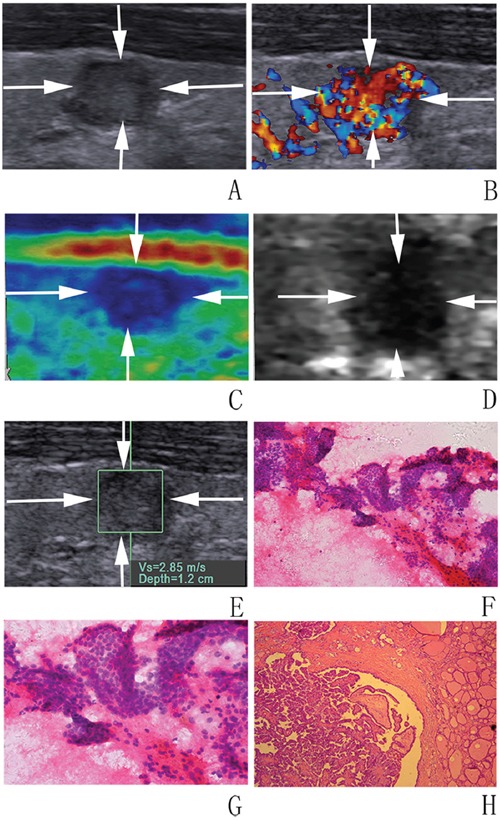
Images in a 32-year-old woman with papillary mirocarcinoma **A, B. at conventional US, an 8-mm thyroid nodule (arrows) in the right lobe of the thyroid appears to have moderate hypoechogenicity, poorly defined margin, irregular shape, and rich blood flow**. At elastography, **C.** CSE score of 4 (arrows), **D.** ARFI imaging grade of VI (arrows), and **E.** SWS of “2.85” m/s are assigned. **F, G.** FNA cytology (haematoxylin-eosin stain, original magnification, ×200 (F), ×400 (G)) shows part follicle cells with nuclear atypia and the nodule is diagnosed as AUS/FLUS. **H.** Histologic specimen (hematoxylin-eosin stain; original magnification, ×50) shows that this thyroid nodule is finally confirmed to be a papillary mirocarcinoma.

**Figure 3:**
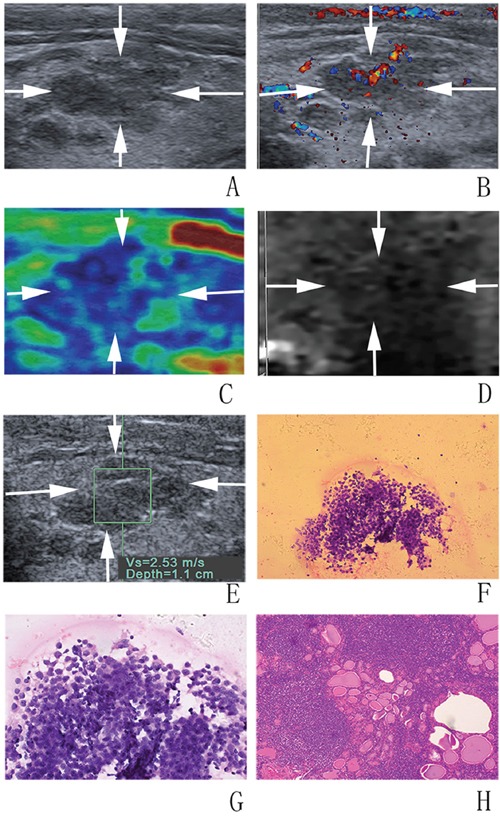
Images in a 61-year-old woman with Hashimoto nodule **A, B. at conventional US, a 12-mm thyroid nodule (arrows) in the right lobe of the thyroid appears to have coarse echogenicity background, moderate hypoechogenicity, poorly defined margin, irregular shape, and rich blood flow.** At elastography, **C.** CSE score of 3 (arrows), **D.** ARFI imaging grade of III (arrows), and **E.** SWS of “2.53” m/s are assigned. **F, G.** FNA cytology (haematoxylin-eosin stain, original magnification, ×200 (F), ×400 (G)) shows a few follicle cells with architectural atypia under the background of lymphocytes and diagnosed as AUS/FLUS. **H.** Histologic specimen (hematoxylin-eosin stain; original magnification, ×100) shows that this thyroid nodule is finally confirmed to be a Hashimoto nodule.

In univariate analysis, nodule size was significantly associated with malignancy in which malignant nodules were smaller than benign ones (9.2 ± 3.9 mm vs. 11.8 ± 6.1 mm; *P* = 0.024). Conversely, patient sex, age, nodule position and thyroid background did not achieve significant differences (all *P* > 0.05). As to conventional US, halo sign, echogenicity, nodule component, shape, height and width, margin, and calcification, were associated with malignancy (all *P* < 0.05) (Figure 2 and Figure 3). In the sub-analysis for nodules 5–10 mm, echogenicity, margin, height and width, and calcification had statistical significances (all *P* < 0.05). For nodules > 10 mm, nodule size, echogenicity, calcification and height and width had statistical differences (all *P* < 0.05) (Table 1). The differences were significant between malignant and benign nodules for CSE score, ARFI imaging grade, SWS and SWS ratio (all *P* < 0.05) (Table 2, Figure 2 and Figure 3)

**Table 1:** Basic characteristics of the patients and US features of the nodules

Characteristics	Overall	Malignant	Benign	*P* value	nodules 5–10 mm (n=48)		*P* value	nodules>10mm (n=55)		*P* value
					Malignant	Benign		Malignant	Benign	
Number of patients	103	38	65		21	27		17	38	
Sex (female/male)	89/14	34/4	55/10	0.488	19/2	24/3	1.000	15/2	31/7	0.824
Mean age (y)	56±11	54±14	57±9	0.214	52±15	56±10	0.294	55±13	57±8	0.496
Range	25-76	25-76	31-70		25-72	31-69		25-76	40-70	
Number of nodules	103	38	65		21	27		17	38	
Position				0.546			1.000			0.403
Left/isthmus/right	47/5/51	16/3/19	31/2/32		9/2/10	11/2/14		7/1/9	20/0/18	
Mean size (mm)	10.8±5.5	9.2±3.9	11.8±6.1	0.024*	6.3±1.6	6.9±1.7	0.196	12.9±2.5	15.2±5.7	0.041*
Range	5-35	5-18	5-35		5-10	5-10		11-18	11-35	
Background				0.062			0.675			0.126
Homogenous / coarse	83/20	27/11	56/9		16/5	23/4		11/6	33/5	
Halo sign				0.023*			0.435			0.095
Absent/present	73/30	32/6	41/24		19/2	21/6		13/4	20/18	
Echogenicity				0.000*			0.000*			0.008*
Hyper-/iso-/hypo-/ marked hypo-/mixed-	1/12/51/23/16	0/0/18/19/1	1/12/33/4/15		0/0/7/14/0	0/6/16/2/3		0/0/11/5/1	1/6/17/2/12	
Shape				0.009*			0.069			0.155
Regular / irregular	26/77	4/34	22/43		1/20	8/19		3/14	14/24	
Margin				0.006*			0.039*			0.155
Well/poorly defined	27/76	4/34	23/42		1/20	9/18		3/14	14/24	
Capsule contact				0.384			0.840			0.363
No contact/ ≤25%/26%–50% / >50%	33/39/22/9	14/15/8/1	19/24/14/8		8/10/3/0	13/10/3/1		6/5/5/1	6/14/11/7	
Internal nodule component				0.013*			0.246			0.160
Solid nodules/ ≤25%/26%–50%/ >50%	87/14/2/0	37/1/0/0	50/13/2/0		21/0/0/0	24/3/0/0		16/1/0/0	26/10/2/0	
Calcification				0.000*			0.009*			0.014*
No-/macro-/micro-	52/38/13	12/24/2	40/14/11		7/14/0	17/7/3		5/10/2	23/7/8	
Height and width				0.000*			0.000*			0.003*
<1 / ≥1	67/36	13/25	54/11		3/18	18/9		10/7	36/2	
Vascularity				0.290			0.392			0.724
Type I/II/III	23/50/30	8/22/8	15/28/22		6/12/3	9/10/8		2/10/5	6/18/14	

* Indicate the differences are statistically significant.

n number ; US Ultrasound;

**Table 2:** CSE and ARFI elastography between malignant and benign nodules

Characteristics	Overall nodules (n=103)	Malignant nodules (n=38)	Benign nodules (n=65)	*P* value	nodules 5–10 mm		*P* value	nodules >10mm		*P* value
					Malignant (n=21)	Benign (n=27)		Malignant (n=17)	Benign (n=38)	
CSE				0.007*			0.090			0.038*
CSE score 1	7	1	6		1	1		0	5	
CSE score2	30	5	25		4	14		1	11	
CSE score 3	53	24	29		11	9		13	20	
CSE score 4	13	8	5		5	3		3	2	
ARFI imaging				0.000*			0.000*			0.000*
Grade I	3	0	3		0	1		0	2	
Grade II	23	0	23		0	7		0	16	
Grade III	29	3	26		3	13		0	13	
Grade IV	31	20	11		9	5		11	6	
Grade V	14	12	2		6	1		6	1	
Grade VI	3	3	0		3	0		0	0	
pSWE										
SWS (m/s)	2.62±1.34	3.36±1.76	2.19±0.74	0.000*	3.27±1.51	2.16±0.75	0.005*	3.47±2.08	2.22±0.73	0.027*
Range	0.12-8.4	1.16-8.4	0.12-3.46		1.57-7.58	0.12-3.22		1.16-8.4	0.12-3.46	
SSWS (m/s)	2.22±0.54	2.14±0.61	2, 27±0.49	0.277	2.09±0.63	2.31±0.56	0.208	2.20±0.61	2.24±0.43	0.801
Range	0.76-3.63	0.76-3.45	1.19-3.63		0.76-3.06	1.34-3.63		1.28-3.45	1.19-3.15	
SWS ratio	1.26±0.82	1.71±1.14	0.99±0.37	0.000*	1.74±1.19	0.95±0.32	0.007*	1.67±1.11	1.02±0.40	0.030*
Range	0.05-5.44	0.51-5.44	0.05-2.24		0.76-5.44	0.06-1.75		0.51-4.74	0.05-2.44	

* Indicate the differences are statistically significant.

n, number; CSE, Conventional strain elasticity; ARFI imaging, acoustic radiation force impulse imaging; pSWE, point shear wave elastography; SWS, shear wave speed; SSWS, surrounding shear wave speed; SWS ratio, shear wave speed ratio

### Multivariate logistic regression analysis

Multivariate logistic regression analysis showed that ARFI imaging grade ≥ 4 (OR: 3.375; 95% CIs: 2.098-5.431) was the most significant independent predictor, followed by marked hypoechogenicity (OR: 2.205; 95% CIs: 1.293-3.761) (Table 3).

**Table 3:** Multivariate logistic regression analysis for predicting malignant nodules

Characteristics	OR	95% CIs	*P* value
Marked hypoechogenicity	2.205	1.293-3.761	0.004
ARFI imaging grade ≥ 4	3.375	2.098-5.431	0.000

ARFI imaging, acoustic radiation force impulse imaging; OR, odd ratio; CIs, Confidence intervals

### Diagnostic performance

The US features of irregular shape, poorly defined margin and absence of halo sign had relatively high sensitivities (89.5%, 89.5%, and 84.2%, respectively) whereas poor specificities (33.8%, 35.4%, and 36.9%, respectively); marked hypoechogenicity had a high specificity (93.8%) whereas a poor sensitivity (50.0%) (Table 4).

**Table 4:** The diagnostic performance for US features, CSE, ARFI elastography and combined ARFI elastography

		Sensitivity (%)	Specificity (%)	PPV (%)	NPV (%)	Accuracy (%)	Az (95 % CIs)
US feature							
	Moderate hypoechogenicity	47.4% (18/38)	49.2% (32/65)	35.3% (18/51)	61.5% (32/52)	48.5% (50/103)	0.483 (0.367-0.599)
	Marked hypoechogenicity	50% (19/38)	93.8% (61/65)	82.6% (19/23)	76.3% (61/80)	77.7% (80/103)	0.719 (0.622-0.803)
	Irregular shape	89.5% (34/38)	33.8% (22/65)	44.2% (34/77)	84.6% (22/26)	54.4% (56/103)	0.617 (0.516-0.711)
	Poorly defined margin	89.5% (34/38)	35.4% (23/65)	44.7% (34/76)	85.2% (23/27)	55.3% 57/103	0.624 (0.523-0.718)
	Absence of halo sign	84.2% (32/38)	36.9% (24/65)	43.8% (32/73)	80% (24/30)	54.4% (56/103)	0.606 (0.505-0.701)
	Microcalcification	63.2% (24/38)	78.5% (51/65)	63.2% (24/38)	78.5% (51/65)	72.8% (75/103)	0.708 (0.610-0.794)
	Height/width≥1	65.8% (25/38)	83.1% (54/65)	69.4% (25/36)	80.6 (54/67)	76.5 (79/103)	0.744 (0.649-0.825)
CSE							
	CSE score≥3	84.2% (32/38)	47.7% (31/65)	48.5% (32/66)	83.8% (31/37)	61.2% (63/103)	0.660 (0.560-0.750)
ARFI elastography							
	ARFI imaging grade≥4	92.1% (35/38)	80.0% (52/65)	72.9% (35/48)	94.5% (52/55)	84.5% (87/103)	0.861 (0.748-0.900)
	SWS>2.94 m/s	52.6% (20/38)	92.3% (60/65)	80% (20/25)	76.9% (60/78)	77.7% (80/103)	0.725 (0.628-0.808)
	SWS ratio>1.09	76.3% (29/38)	70.8% (46/65)	60.4% (29/48)	83.6% (46/55)	72.8% (75/103)	0.735 (0.639-0.817)
Combined ARFI							
	b+c	52.6% (20/38)	95.4% (62/65)	87.0% (20/23)	77.5% (62/80)	79.6% (82/103)	0.740 (0.644-0.822)
	a+b	47.4% (18/38)	100% (65/65)	100% (18/18)	76.5% (65/85)	80.6% (83/103)	0.737 (0.641-0.819)
	a+c	68.4% (26/38)	96.9% (63/65)	92.9% (26/28)	84% (63/75)	86.4% (89/103)	0.827 (0.740-0.894)
	b/c	76.3% (29/38)	67.7% (44/65)	58% (29/50)	83.0% (44/53)	70.9% (73/103)	0.720 (0.623-0.804)
	a+ b/c	76.3% (29/38)	100% (65/65)	100% (29/29)	87.8% (65/74)	91.3% (94/103)	0.882 (0.803-0.937)

PPV positive predictive value; NPV negative predictive value; Az Area under the curve; CIs confidence intervals

a: ARFI imaging grade≥4 ; b: SWS>2.94 m/s; c: SWS ratio>1.09

The best cut-off values for CSE score, ARFI imaging grade, SWS and SWS ratio were score 3, grade 4, 2.94 m/s and 1.09, respectively. ARFI imaging (Az: 0.861; 95% CIs: 0.748 - 0.90) had the highest diagnostic performance in comparison with conventional US (Az: 0.606 - 0.744; 95% CIs: 0.505 - 0.825), CSE (Az: 0.660; 95% CIs: 0.560 - 0.750), SWS (Az: 0.725; 95% CIs: 0.628 - 0.808) and SWS ratio (Az: 0.735; 95% CIs: 0.639 - 0.817). The combination of ARFI imaging with SWS or SWS ratio measurement increased the specificity from 80.0% to 100% and positive predictive value (PPV) from 72.9% to 100% respectively, in comparison with ARFI imaging alone (both *P* < 0.05) (Table 4).

### Analysis on misdiagnosis

Hashimoto nodule (n=4) and calcification (n=5) accounted for 69% (9/13) of 13 false positive results on ARFI imaging and all the 3 (100%) false negative results were papillary thyroid microcarcinomas (PTMCs). For the combination method, all (100%) the 9 false negative results were solid nodules, 7 (77.8%) were irregular shape, 7 (77.8%) were poorly defined margin. No false positive results were found.

## DISCUSSION

The malignancy risk of AUS/FLUS nodules is varying and uncertain, therefore the recommended treatment of AUS/FLUS nodules is usually diagnostic thyroid lobectomy or repeat FNAC [26, 27]. However, most of these nodules are benign at final pathological examinations after surgery or remain as AUS/FLUS nodules on repeat FNAC [28]. Risk stratification of these nodules may reduce unnecessary invasive procedures or avoid possible complications.

In the present study, AUS/FLUS nodules were found in 11.8% nodules after FNAC and its malignancy rate was 36.9%. Clinical features such as larger nodule size, male and age > 40 years were reported to increase the probability of malignancy for AUS/FLUS nodules [2, 13, 29]. However, Gweon et al. [4] discovered that sex, nodule size and age were not associated with malignancy. In the current study, malignant nodules were statistically smaller, which was related to the high proportion of microcarcinomas. Many other reports had emphasized the importance of US features in evaluating the AUS/FLUS nodules [2, 12, 13, 18, 30]. Mendez et al. [30] suggested that hypoechogenicity, irregular margin, microcalcification, and taller-than-wide shape were significantly associated with malignancy, whereas Samir et al. [31] reported that no B-mode US or Doppler characteristics displayed significant differences between benign and malignant indeterminate nodules. Our results found that only marked hypoechogenicity on conventional US was independent factor for malignancy. The differences might be due to different sample size of the AUS/FLUS nodules. In addition, it was reported that specimens diagnosed as AUS/FLUS were associated with the highest discordance rates among different centers [9]. Also, relatively high intra-observer variability in this difficult diagnostic category was documented [9].

Recent studies focused on the ability of US elastography for differentiation between benign and malignant AUS/FLUS nodules, whereas the results were inconsistent [16, 20, 32]. In the current study, the best cut-off values for CSE score, ARFI imaging grade, SWS and SWS ratio were score 3, grade 4, 2.94 m/s and 1.09, respectively. The sensitivity, specificity and Az for CSE score ≥ 3 were 84.2%, 47.7% and 0.660 (95% CIs: 0.560 – 0.750), respectively and CSE was failed to be identified as a predictor. For point SWS measurement including SWS and SWS ratio, the sensitivities, specificities and Azs were 52.6% - 72.3%, 70.8% - 92.3% and 0.725 - 0.735. In another study, Samir et al [31] indicated that SWS imaging may be a useful tool in preoperative malignancy risk assessment of follicular-patterned thyroid nodules, with a cut-off median value of 22.30 kPa for Young modulus, which had a higher sensitivity (82%), specificity (88%) and Az (0.81) than our results [31]. The underlying reason is that our findings are specific to particular patient cohorts with AUS/FLUS results, while the previous study focused on the follicular-patterned thyroid nodules. In addition, in the present study ARFI elastography was performed in longitudinal plane to avoid possible influencing factors such as pulsation of carotid artery and trachea, while SWS imaging in Samir's study were obtained in the transverse plane as optional plane [31]. Woo et al. [33] thought the SWS measured with ARFI and SWS imaging may have systemic differences, which may complicate the direct comparison between them.

ARFI imaging belongs to strain imaging in nature, which reduces the interference of man-made factors and improves the reproducibility of the operation. Previous studies confirmed the usefulness of ARFI imaging in diagnosing thyroid nodules and focal liver lesions [34–36]. In the current study, ARFI imaging was the most significant independent predictor for malignancy by multivariate analysis. ARFI imaging grade (Az: 0.861) had higher diagnosis performance compared other single risk features including point SWS measurement (Azs: 0.606 - 0.744). The result might be ascribed to the fact that ARFI imaging reflects the stiffness of the entire target nodule, whereas point SWS measurement reveals tissue elasticity in selected region in the lesion. In addition, it was difficult to avoid the microcalcification and cystic areas when the microcalcifications were diffuse or the cystic areas were small and indistinguishable, which might lead to an inaccurate SWS measurement. Therefore, ARFI imaging may play more promising role in clinical practice. However, ARFI imaging also could be confounded by several factors, including inflammation and calcification, both of which are possible to increase estimated tissue stiffness [37, 38].

Sporea et al [39] reported combining two elastographic methods could obtain a high specificity (93.3%) and PPV (96.8%) for predicting significant liver fibrosis. Therefore we also tried to use the combined elastography feature to evaluate the Bethesda category III nodules. As combination of ARFI imaging with point SWS measurement (SWS or SWS ratio), both the specificity and PPV significantly increased. The increase of specificity and PPV is meaningful, which indicates combining ARFI technique is helpful to make definite diagnosis for both benign and malignant nodules. However, the value of elastography in evaluating thyroid nodules remains to be determined. Russ [40] speculated about the classification of thyroid carcinomas into two categories. Irregular infiltrative carcinomas harbor a fibrous component with the low elasticity and elastography can be applied to detect this. Non-infiltrative carcinomas with a regular shape and borders have high elasticity, so elastography contribute little to their evaluation.

There were several limitations in the study. Only patients who underwent surgery were enrolled, which might lead to selection bias. However, at the current stage only pathological examination can be used as the reference standard. Repeat FNAC or follow-up is inadequate to exclude or confirm malignancy for Bethesda category III nodules. Furthermore, 36.9% of the nodules in this series were malignant at surgery. It was beyond the recommended malignancy rate. It is possible that the high-risk Bethesda III nodules may be more likely to be triaged to surgery and the reproducibility of interpreting AUS/FLUS is also limited [9]. In literatures, the malignancy risk of Bethesda III nodules was varying from 5% to 48% [9, 12–14], which indicates that the Bethesda category should be independently defined at each center to guide clinicians for risk estimation. On the other hand, the current study involved some nodules that were less than 10 mm in diameter. According to the 2015 American Thyroid Association (ATA) guideline, those nodules < 10 mm require evaluation because of associated lymphadenopathy, suspicious US findings, location close to recurrent laryngeal nerve or trachea, or other high-risk clinical factors such as a family history of thyroid cancer or a childhood history of head and neck irradiation. A small percentage of PTCs < 10 mm present with clinically significant regional or distant metastases and signs of progression during follow-up [9]. For point SWS measurement in evaluating nodules smaller than 10mm, it is possible that the peripheral thyroid parenchyma is also included in the ROI box. In addition, the case number was relatively small, thus future studies with large case series are needed. Finally, it was a single-center experience and future multi-center studies are mandatory.

In summary, the present study demonstrates that ARFI elastography is a promising tool for preoperative malignancy risk stratification of patients with AUS/FLUS nodules. Specifically, combination of ARFI imaging with point SWS measurement could significantly improve specificity and PPV. ARFI elastography may provide an easy and highly efficient way to help clinician to make correct decision for Bethesda category III nodules with regard to subsequent treatment planning.

## MATERIALS AND METHODS

This study was approved by the Ethics Committee of the Shanghai Tenth People's Hospital and informed consent was obtained from all the patients. The study was performed in accordance with relevant guidelines and regulations.

### Study population

From June 2013 to August 2015, we prospectively examined 4650 consecutive patients with 5260 thyroid nodules with conventional US, CSE, and ARFI elastography in the tertiary hospital. Of them, a total of 3300 consecutive patients with 3940 thyroid nodules were subject to US-guided FNAC to rule out malignancy. Cytological results found 466 (11.8%, 466/3940) AUS/FLUS nodules in 462 patients after FNAC. Of all the AUS/FLUS nodules that had elastography performed, patients were enrolled on the basis of the inclusion criteria as following: (a) Thyroid nodules were visible on conventional US. (b) At least 5 mm in maximal diameter. (c) With pathological confirmation by surgery. At last, pathological confirmation was obtained in 115 nodules in 114 patients. Twelve AUS/FLUS nodules in 11 patients were excluded due to image incompleteness. Finally, 103 AUS/FLUS nodules in 103 patients were included for analysis. The 103 subjects included 89 females and 14 males, with an overall mean age ± standard deviation of 56 ± 11 years (age range: 25 – 76 years).

### Conventional US, CSE and ARFI elastography examinations

All the examinations were performed with the same S2000 US scanner (Siemens Medical Solutions, Mountain View, Calif, USA) with the 7-17 MHz and/or 4-9 MHz linear transducer for conventional US and the 4-9 MHz linear transducer for elastography and were performed by 1 of 2 board-certified radiologists with more than 9 years' experience in thyroid US and 5 years of experience in thyroid elastography.

The patients were scanned in supine position with dorsal flexion of the head. Conventional transverse and longitudinal US images were obtained for each target nodule firstly. To obtain optimal images, the target nodule was placed at the center of the screen and the machine settings were constantly adjusted. The US features were evaluated and recorded. The precise location of nodule within the thyroid lobe was also recorded, such as upper, middle, or lower portion of the lobe. The distance from the thyroid capsule, carotid artery or trachea was also recorded, which facilitated correlation between US and pathology results for heterogeneous glands containing multiple coalescent nodules. Afterwards, CSE and ARFI elastography were performed in the longitudinal direction of the thyroid nodule to avoid possible influencing factors such as pulsation of carotid artery. The sampling box for CSE and ARFI imaging were selected to contain the target nodule and sufficient surrounding thyroid tissue. CSE was performed with a light pressure, with the quality indicator value above 60 to ensure sufficient quality image. ARFI elastography was thereafter initiated, with the patient holding the breath for a few seconds. ARFI involves ARFI imaging (i.e. virtual touch tissue imaging [VTI]) and point SWS measurement (i.e. virtual touch tissue quantification [VTQ]) for targeting an anatomic region to interrogate the elastic properties. Tissue within the sampling box or shear wave (SW) ROI is mechanically excited by using short-duration acoustic pulses to generate localized displacements, which results in the propagation of transverse shear waves. ARFI imaging shows the elasticity of tissue (i.e. the longitudinal displacement) with gray-scale image in which the brightness means decreased tissue stiffness whereas the darkness indicates increased tissue stiffness. Point SWS measurement could assess the tissue elasticity quantitatively. The SWS is obtained by scaling the time to peak displacement at each lateral location and is shown on the screen automatically. The SW ROI size is fixed at 6 mm × 5 mm. The basic principles for SW ROI selection are as follows: (1) The SW ROI is placed on the solid portion of the nodule; (2) The calcified or cystic portions are avoided. (3) The SW ROI is placed on the peripheral portion of the nodule to avoid possible necrotic tissue. (4) Adjacent thyroid tissue is not included. The SWS measurement was repeated for 7 times without movement of the transducer. Afterward, the SW ROI was moved to the relatively homogeneous surrounding thyroid tissue (usually more than 5 mm from the nodule) at the same depth and the surrounding shear wave speeds (SSWSs) were also repeatedly measured 7 times at the same site.

### Image interpretation

All images of US, CSE and ARFI imaging were scored and recorded independently by two experienced investigators with more than 10 years of experience in thyroid US and 5 years of experience in thyroid elastography, who were blind to the patients' identities and pathological diagnoses. All investigators were trained before analyses with the same standard. A training process was carried out in 30 extra patients before the study until high observer consistency (Kappa values > 0.6) was obtained. When discordance appeared for the evaluation between the two investigators, another senior investigator with more than 20 years of experience in thyroid US and 7 years of experience in thyroid elastography reviewed the images and made the final decision.

On gray-scale US, the target nodule was evaluated for size (largest diameter, subgrouped as 5–10 mm or > 10 mm), position (left, isthmus or right lobe), thyroid background (homogenous or coarse echogenicity background), halo sign (present or absent), capsule contact (no contact, less than 25%, 26% – 50% or more than 50% of the perimeter in contact with the capsule), echogenicity (hyper-, iso-, hypo-, marked hypo-, or mixed echogenicity, compared with surrounding thyroid tissue or nearby strap muscle), internal nodule component (four categories: completely solid, cystic portion ≤ 25%, cystic portion 26% – 50%, or cystic portion > 50%), shape (regular or irregular), margin (well or poorly defined), calcification (microcalcification ≤ 1.5 mm in diameter, macrocalcification > 1.5 mm in diameter with acoustic shadow, or no calcification), height and width (taller than wide or wider than tall). Color Doppler US patterns were defined as absence of visible blood flow (type I), peripheral blood flow and absent or slight internal blood flow (type II), and rich internal blood flow and absent or slight peripheral blood flow (type III) [24].

The CSE score was displayed that was based on a color scale ranging from red color (soft component) to blue color (hard component) over the B-mode image. The CSE was classified into four patterns according to Asteria et al. [19]: score 1, prevalence of red and green color in nodule; score 2, predominant green with few blue areas/spots in nodule ; score 3, predominant blue with few green areas/spots in nodule ; score 4, the nodule is displayed entirely in blue.

The images of ARFI imaging were thereafter divided into grade I to grade VI as following [23]: Grade I, predominantly white for the whole nodule; Grade II, predominantly white with few black portions; Grade III, equal black and white portions; Grade IV, predominantly black with a few white spots; Grade V, almost completely black, and Grade VI, entirely black. Higher grades mean stiffer tissue.

For point SWS measurement, the investigators just read the SWS measurement results in the static images retrieved from hard disk. The highest and the lowest values of SWS and SSWS were eliminated and the mean of the rest 5 measurements was calculated. SWS ratio was figured out by mean intra-nodular and extra-nodular SWS values. After excluding other factors such as movement or breath of patient, inappropriate ROI placement or improper precompression, the measurement results of “X.XX m/s” (either extremely soft or extremely hard) were displaced by 0 m/s (the cystic portion) or 8.4 m/s (the solid portion) as suggested by previous studies [22, 25].

### US-guided FNAC procedure and cytopathological classification

US-guided FNAC was performed using a 22-gauge PTC needle (Hakko, Nagano, Japan) under local anesthesia. Three to five smears were obtained for each target nodule, which were collected in 95% alcohol and were submitted for haematoxylin-eosin stain. All cases were reported using a six-tiered diagnostic system with BSRTC as follows: (1) nondiagnostic or unsatisfactory (Bethesda category I), (2) benign (Bethesda category II), (3) AUS/FLUS (Bethesda category III), (4) follicular neoplasm or suspicious for follicular neoplasm (Bethesda category IV), (5) suspicious for malignancy (Bethesda category V), and (6) malignant (Bethesda category VI) [6]. All FNAC were reported by one of three cytopathologists with more than 3 years of experience in thyroid cytopathology. When FNAC report was Bethesda category III, another 2 senior cytopathologists, with more than 20 years of experience in thyroid pathology and 6 years of experience in thyroid cytopathology, reviewed the slides and made the final decision with consensus. Each patient was precisely positioned for target nodule in US before surgery. AUS/FLUS nodules were identified by correlating cytology reports with US and pathology reports.

### Statistical analysis

All the statistical analyses were performed using the SPSS software (version 19.0, Chicago, IL, USA) and MedCalc software (version 15.2, Mariakerke, Belgium). Continuous variables were compared by independent two-sample *t* test, while Chi-square test or Fisher's exact test was used to analyze the categorical variables. A step-wise multivariate logistic regression analysis was performed to find the independent predictors for malignancy. Receiver operating characteristic (ROC) curve analysis was performed to assess the diagnostic performance. The comparisons of the area under the ROC curves (Azs) were performed by Z test. The optimal cut-off value for each variable, as well as the corresponding sensitivity and specificity, was obtained from ROC analysis when Youden index was maximum. The PPV, negative predictive value (NPV) and accuracy were calculated by the diagnostic test 2×2 contingency tables. Sub-analysis was performed according to nodule size (5–10 mm and > 10 mm). About the combining methods, “Or” is defined as either of two methods diagnosed the nodule as malignant, which is consider as malignant; “And” is defined as both methods diagnosed the nodule as malignant at the same time, which is considered as malignant. A two-tailed P value < 0.05 was considered to be statistically significant. Confidence intervals (CIs) were recorded as two-sided exact binomial 95% CIs.
